# Supplementary material for: SSA4 Mediates Cd Tolerance via Activation of the Cis Element of VHS1 in Yeast and Enhances Cd Tolerance in Chinese Cabbage
Source: Int J Mol Sci. 2024 Oct 14;25(20):11026. doi: 10.3390/ijms252011026 (PMC11507436; doi:10.3390/ijms252011026)
Supplement: Supplementary file 1 [file ijms-25-11026-s001.zip › Supplementary figure.pptx]

## Slide 1
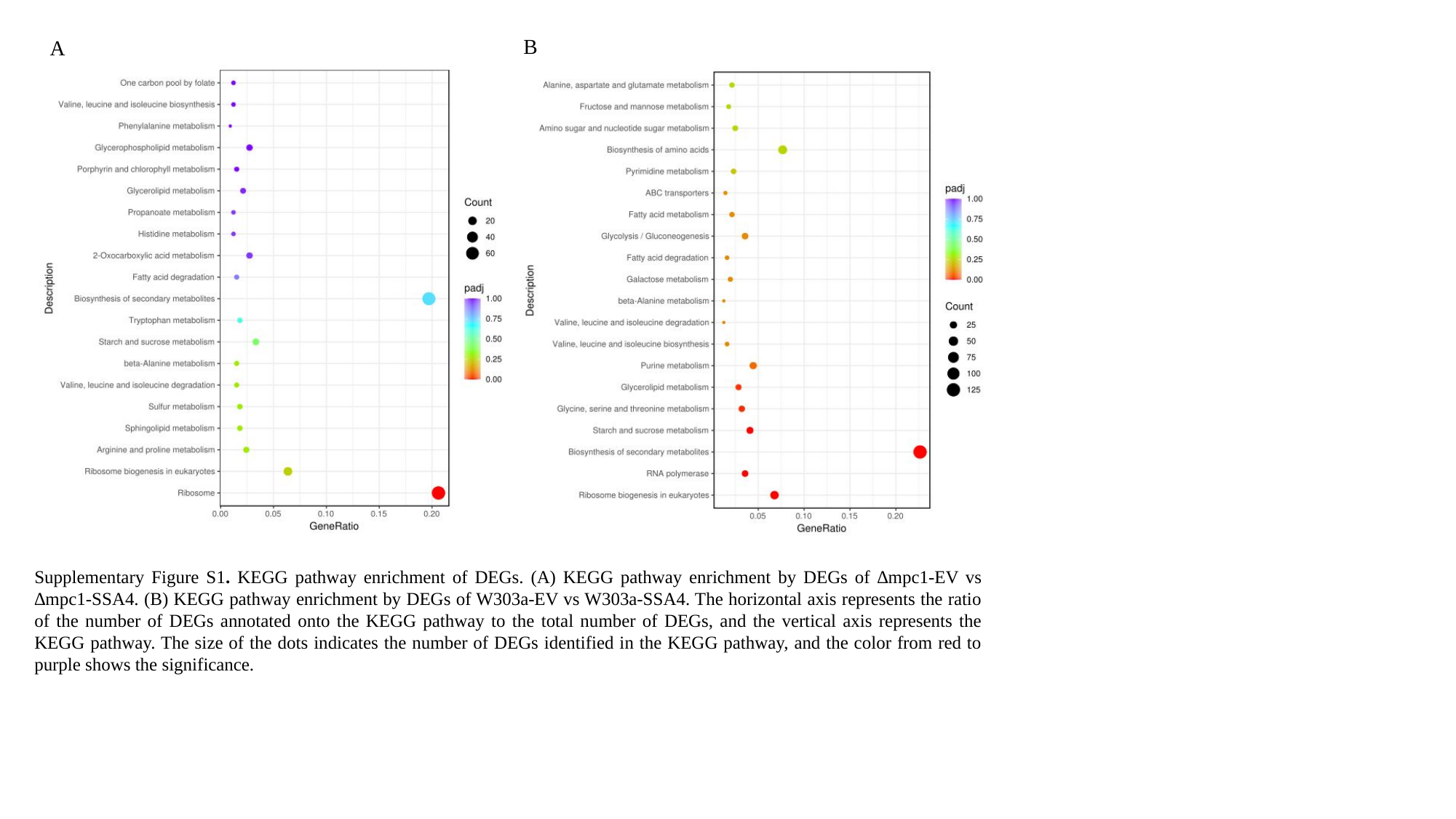

B
A
Supplementary Figure S1. KEGG pathway enrichment of DEGs. (A) KEGG pathway enrichment by DEGs of ∆mpc1-EV vs ∆mpc1-SSA4. (B) KEGG pathway enrichment by DEGs of W303a-EV vs W303a-SSA4. The horizontal axis represents the ratio of the number of DEGs annotated onto the KEGG pathway to the total number of DEGs, and the vertical axis represents the KEGG pathway. The size of the dots indicates the number of DEGs identified in the KEGG pathway, and the color from red to purple shows the significance.
