# Supplementary material for: SSA4 Mediates Cd Tolerance via Activation of the Cis Element of VHS1 in Yeast and Enhances Cd Tolerance in Chinese Cabbage
Source: Int J Mol Sci. 2024 Oct 14;25(20):11026. doi: 10.3390/ijms252011026 (PMC11507436; doi:10.3390/ijms252011026)
Supplement: Supplementary file 1 [file ijms-25-11026-s001.zip › Supplementary Table S1.pdf]

Supplementary Table S1. Chinese cabbage BrSSA4 gene family. BrSSA4a to m were assigned according to their distribution in the genome. The GC content, number of amino acids, molecular weight (MW) and theoretical isoelectric point (pI) were calculated by DNASTAR (Madison, WI, USA). The rest data were downloaded from Brassica Database (http://www.brassicadb.cn/).

Gene	Accession no.	Chr. (strand)	Start/stop codon	CDS (bp)	GC content (%)	Length * (aa)	MW * (Daltons)	pl *
BrSSA4a	*BraA01g019900.3C*	A01 (-)	10687460/10691347	2685	44.28	895	99972.36	6.03
BrSSA4b	*BraA01g038810.3C*	A01 (+)	26269513/26272150	1953	51.51	651	71270.78	5.05
BrSSA4c	*BraA02g003050.3C*	A02 (+)	1492088/1495420	2043	49.24	681	72870.43	5.84
BrSSA4d	*BraA03g047170.3C*	A03 (+)	23836812/23840680	2631	44.81	877	97595.80	5.87
BrSSA4e	*BraA04g004290.3C*	A04 (-)	2645163/2648333	2373	49.81	791	87162.06	5.19
BrSSA4f	*BraA05g012280.3C*	A05 (-)	6664594/6666288	1695	53.69	565	60818.93	5.40
BrSSA4g	*BraA06g001280.3C*	A06 (+)	802745/805920	2457	49.12	819	90298.65	5.11
BrSSA4h	*BraA06g008520.3C*	A06 (+)	4681135/4681341	207	52.17	69	7785.94	11.52
BrSSA4i	*BraA06g012060.3C*	A06 (-)	6488327/6490270	1944	50.82	648	71042.60	5.47
BrSSA4j	*BraA07g008590.3C*	A07 (-)	8612041/8614425	2010	49.80	670	73847.57	5.00
BrSSA4k	*BraA08g020460.3C*	A08 (-)	15677635/15680441	2154	48.33	718	76421.04	5.04
BrSSA4l	*BraA08g021750.3C*	A08 (-)	16460538/16462970	2031	50.42	677	72488.42	5.83
BrSSA4m	*BraA08g029700.3C*	A08 (+)	20365638/20367590	1953	50.08	651	71341.70	5.22

* Length, WM and pI refer to the translated BrSSA4 proteins.
