# Supplementary material for: SSA4 Mediates Cd Tolerance via Activation of the Cis Element of VHS1 in Yeast and Enhances Cd Tolerance in Chinese Cabbage
Source: Int J Mol Sci. 2024 Oct 14;25(20):11026. doi: 10.3390/ijms252011026 (PMC11507436; doi:10.3390/ijms252011026)
Supplement: Supplementary file 1 [file ijms-25-11026-s001.zip › Supplementary Table S2.pdf]

Supplementary Table S2. qRT-PCR primer sequences.

Name	Forward Primer (5'-3')	Reverse Primer (5'-3')
Sc680-RT	CAAACAACTGCCAAGTGTTATG	GCTTATCATGAACTGTTCTTGGA
Sc1620-RT	ACGTGACTTCAGAAAGCATGG	TAGACGACACCGGTCTTACC
Sc1667-RT	TGGCGGTTCTTCAAATGAGT	AGCATGGATTCCTCAAGACA
Sc2938-RT	TGAAACTGTATTGCAACTGAGAAATG	TGCTGGATCCCAATCATCCTGT
Sc2995-RT	AGACCAAGAGAAGTACGACA	GCTCTGGTGTAGATAGCTTGC
Sc4071-RT	GATAATGGCAGTCGCAGTGG	TCGTTATCGGACTCGCAAGA
Sc4082-RT	GGTGATGAAAGGCGACAAGG	ACCAGCCCTTTGAAGACGTA
Sc4516-RT	ATGACGACATTTCCGCCTTG	TGGCATATCACACCTTGGGT
Sc4598-RT	TGTCTGCTGGTTGATTGCTG	GGCCTTGACCAGGTTGAATG
ScActin-RT	CACGGATAGTGGCTTTGGTGAACAATTAC	TATGATTATCTGGCAGCAGGAAAGAACTTGGG
P4071-F1-RT	GCCATCGGAAGATAAACGC	TTATGAAGGAGGAGACGGAGA
P4071-F2-RT	ATGCACGGATCAGTTCACG	TACTCTTGGGTCGGTCAGG
P4071-F3-RT	GCCATCGGAAGATAAACGC	TTATGAAGGAGGAGACGGAGA
P4071-F4-RT	CAGTTACGTTAAAGGGACCAA	GGACGCGGACCTAGATACA
P4071-F5-RT	CGCTACCATTGTTTTTTTG	TTCTTTCTCTTATTTTATTTTACTT
BrSSA4a-RT	GGGACTCGGGACTTGGAGGA	TGGAGCTGCAGTGTTTGCAC
BrSSA4b-RT	ATCTCCGGCCCAGCAGAGAA	GACAGACGTGCCGAGGAAGG
BrSSA4c-RT	GTTCGACGATCCGCAAACGC	TCGTCAGCACAAACGCTCCA
BrSSA4d-RT	CAAACGTGGGCAGAGTCCGA	CCGTGATCCCTGCAGCTTCC
BrSSA4e-RT	CGGAGAGCGAGCCGCTTAAT	CTCCCTGAACTTGGCCGCAA
BrSSA4f-RT	GCCTCATAGGTCGCGTGGAC	TCGACCTCCACGCGTTGTTC
BrSSA4g-RT	CCCTGTGGCGCCGTTGAATA	GAGCCCAGCATCAGCGAGAG
BrSSA4h-RT	GGTACATGGGCGAGGTGCAG	GCCGCCGCATCCAAATAAGC
BrSSA4i-RT	CAACGACCGCGTGGAGATCA	GTGAGGGTTCAGCGCGACTT
BrSSA4j-RT	TGACGTTGCGCCACTCACTT	AGACGGTGGTCTGCTGGTCT
BrSSA4k-RT	CGTAGCCGCCATGGAAGGAG	GGGTTAACAACCGCCTGCCT
BrSSA4l-RT	TCATCACCGCAGACGCTTCC	TTTGGCACGGACACCTCGTC
BrSSA4m-RT	ACGTCGCCTTCACAGACACC	GGGTCCCGCAACGACTTTGA

BrACT1-RT	CGTACTACCGGTATTGTGCT	GAGCTGGATTTGGAAGTCTC
