# Supplementary material for: SSA4 Mediates Cd Tolerance via Activation of the Cis Element of VHS1 in Yeast and Enhances Cd Tolerance in Chinese Cabbage
Source: Int J Mol Sci. 2024 Oct 14;25(20):11026. doi: 10.3390/ijms252011026 (PMC11507436; doi:10.3390/ijms252011026)
Supplement: Supplementary file 1 [file ijms-25-11026-s001.zip › Supplementary Table S3.pdf]

Supplementary Table S3. Primers for gene clone and vector conduction.

Name	Forward Primer (5'-3')	Reverse Primer (5'-3')
ScSSA4-CDS	ATGTCAAAAGCTGTTGGTATT	CTAATCAACCTCTTCAACCGT
ScPom34-CDS	ATGAAGATTCAGGCGGGC	TTATATTTTCCCCCTTGGGGA
Sc680-CDS	ATGTCACTAAGAAACATATCAATGATC	TTATACTTTTACGTGCTCCGATA
Sc1620-CDS	ATGGGTAAATCACATGGTTAC	TTAAATGAAGGTTTCGTATGG
Sc1667-CDS	ATGAAGAAAACAATTTACAAAGTC	TTAAGTAAAAGAGCTTTTGAAAC
Sc2938-CDS	ATGGCACCGAAAAAGAAATC	TCAAAAGAAAAGGTCTCGAAAAAT
Sc2995-CDS	ATGCCTCCAAAGCAACAAT	TTATTCAGAAGCAGTAGCTCTGG
Sc4071-CDS	ATGATGATGTTCCATAATTGC	TTAGTTAGTTAAAGAAGAGGAACTCAT
Sc4082-CDS	ATGTTAAGACAAGCTACAAAAGC	TTACTTTTCAGCTTCCTCTTC
Sc4516-CDS	ATGAACGATACGCTATCAAG	CTAAGCAGTATCTTCGACAGC
Sc4598-CDS	ATGTCTGATTATGTTGAACTATTGA	TTACTCAGAGTCGGTGGCTA
pRS416-GFP-ScSSA4	aatctagaactagtgATGTCAAAAGCTGTTG	gcagcccgggggatcTATCAACCTCTTCAAC
pRS416-GFP-Sc680	aatctagaactagtgATGTCACTAAGAAACATATC	gcagcccgggggatcTTACTTTTACGTGCTCCGATACAGT
pRS416-GFP-Sc1620	aatctagaactagtgATGGGTAAATCACATGGTTACAGATCTC	gcagcccgggggatcTAATGAAGGTTTCGTATGGAACTGGAGC
pRS416-GFP-Sc1667	aatctagaactagtgATGAAGAAAACAATTTACAAAGTC	gcagcccgggggatcTAGTAAAAGAGCTTTTGAAACCAGTTATC
pRS416-GFP-Sc2938	aatctagaactagtgATGGCACCGAAAAAGAAATC	gcagcccgggggatcTAAAGAAAAGGTCTCGAAAAATGTTCAA
pRS416-GFP-Sc2995	aatctagaactagtgATGCCTCCAAAGCAACAATTATCTAAAG	gcagcccgggggatcTTTCAGAAGCAGTAGCTCTGGTGTAGATAG
pRS416-GFP-Sc4071	aatctagaactagtgATGATGATGTTCCATAATTGCAGAATTA	gcagcccgggggatcTGTTAGTTAAAGAAGAGGAACTCATGC
pRS416-GFP-Sc4082	aatctagaactagtgATGTTAAGACAAGCTACAAAAGCACC	gcagcccgggggatcTCTTTTCAGCTTCCTCTTCAACAAC
pRS416-GFP-Sc4516	aatctagaactagtgATGAACGATACGCTATCAAGCTTTTTAA	gcagcccgggggatcTAGCAGTATCTTCGACAGCTTGCTCTGC
pRS416-GFP-Sc4598	aatctagaactagtgATGTCTGATTATGTTGAACTATTGA	gcagcccgggggatcTCTCAGAGTCGGTGGCTACATCCTCCTCCT

pGBKT7-ScSSA4	ggccgaattcccgggtTCAAAAGCTGTTGGTATTGATTTAG	gcaggtcgacggatcCTAATCAACCTCTTCAACCGTTGGG
pGADT7-Sc864	catcgatacgggatcATGAAGATTCAGGCGGGCCAATTG	cgagctcgatggatcTTATATTTTCCCCCTTGGGGACTG
pDOE01-ScSSA4	aactagtggaggatcTATGTCAAAAGCTGTTGGTATTG	acccacctccggatcTTCCTCCTCCTCCTCCTCCATCAACCTCTTCAACCGTTG
pDOE01-ScSSA4-ScPom34	caatggggtccctacTAATGAAGATTCAGGCGGGCCAATTG	acgtcacgtgactacTTCCTCCTCCTCCTCCTATTTTCCCCCTTGGGGACTGTG
1307-MYC-1308-ScSSA4	tatctagaactagtgGGAGGAGGAGGAGGAGGATCAAAAGCTGTTGGTATTGATTTAG	gcagcccgggggatcCTAATCAACCTCTTCAACCGTTG
1307-FLAG-1308-Sc864	ccaggggcccggatcGGAGGAGGAGGAGGAGGAAAGATTCAGGCGGGCCAATTG	cgtatgggtaggatcTTATATTTTCCCCCTTGGGGAC
BrSSA4b-CDS	CAAAGAATTTGATAATCTAATGGC	CTTAATCAACTTCTTCAATCTTGG
3300-GFP-BrSSA4b	cacgcggtacccgggATGGCGGGTAAAGGCGAAGGTCCAG	cgactctagaggatcTTCCTCCTCCTCCTCCTCCAATCAACTTCTTCAATCTTGG
BrSSA4c-CDS	CCCTTCATAGGACTAAGACGC	TCACTTCTTCACTTCCTCGTAGT
3300-GFP-BrSSA4c	cacgcggtacccgggATGGCTACCGCCGCTCTCCTC	cgactctagaggatcTTCCTCCTCCTCCTCCTCCCTTCTTCACTTCCTCGTAGTC
BrSSA4e-CDS	ATCTTCAGAATGAGTGTGGTC	ATCATCTCTTAGGCACTATCTTC
3300-GFP-BrSSA4e	cacgcggtacccgggATGAGTGTGGTCGGGTTCGAC	cgactctagaggatcTTCCTCCTCCTCCTCCTCCGGCACTATCTTCGGTGGGCTTC
BrSSA4h-CDS	ATGCAAATAAATTTGTTTCCTTC	TCAATCAAGCTCCATATCATC
3300-GFP-BrSSA4h	cacgcggtacccgggATGCAAATAAATTTGTTTC	cgactctagaggatcTTCCTCCTCCTCCTCCTCCATCAAGCTCCATATCATC
BrSSA4i-CDS	AATCAACAATGGCGACTAAATC	TTAATCAACCTCTTCTATCTTCGG
3300-GFP-BrSSA4i	cacgcggtacccgggATGGCGACTAAATC	cgactctagaggatcTTCCTCCTCCTCCTCCTCCATCAACCTCTTCTATCTTCGG
